# Psychometric Properties, Factor Structure, and Evidence for Measurement Invariance in the Greek Version of the Disgust Scale-Revised (DS-R)

**DOI:** 10.3390/diseases7020033

**Published:** 2019-04-03

**Authors:** Theodoros Chalimourdas, Silia Vitoratou, Efstathia Matsouka, Dimitra Anna Owens, Leto Kalogeraki, Iraklis Mourikis, Nikolaos Vaidakis, Maria Tzinieri-Kokkosi, Artemios Pehlivanidis, Charalambos Papageorgiou

**Affiliations:** 1 1st Department of Psychiatry, Athens University Medical School, “Eginiton” Hospital Athens Greece, 74 Vas.Sophias Avenue, 11528 Athens, Greece; efi.matsouka@gmail.com (E.M.); dimitra.owens.1990@gmail.com (D.A.O.); letokalogeraki@yahoo.gr (L.K.); irmour@gmail.com (I.M.) vaidanik@med.uoa.gr (N.V.); margkok@med.uoa.gr (M.T.-K.); apechlib@med.uoa.gr (A.P.); chpapag@med.uoa.gr (C.P.); 2 Psychometrics & Measurement Lab, Department of Biostatistics and Health Informatics, Institute of Psychiatry, Psychology & Neuroscience, King’s College London, De Crespigny Park, London SE5 8AF, UK; silia.vitoratou@kcl.ac.uk

**Keywords:** disgust propensity, reliability, validity, bi-factor models, measurement invariance, psychometrics

## Abstract

The Disgust Scale has been designed to measure disgust propensity—the individual ease in experiencing disgust. The present study aimed to explore the validity, reliability, the factor structure, and the measurement invariance of the Greek version of the Disgust Scale-Revised (DS-R). A sample of 754 healthy participants completed the Greek version of the DS-R. A subset (*n* = 363) also completed the revised Symptom checked list and the Eysenck Personality Questionnaire, in order to examine the concurrent validity. Exploratory and Confirmatory factor analyses in different subsets were used to examine the factor structure. Multiple indicators–multiple causes model (MIMIC) models were used to assess the measurement invariance across gender and age. Demographic influences were assessed using *t*-tests, ANOVA, and Pearson’s correlations. Exploratory factor analysis concluded to two and three factor models, with a factor structure similar to the ones proposed in the literature. Confirmatory factor analysis and bi-factor analysis provided evidence in favor of the three-factor solution. Measurement invariance test revealed differences in six items across gender, and three items across age. The psychometric properties of the factors were satisfactory. Demographic influences on the responses were present, especially with respect to gender. The Greek version of the DS-R demonstrated satisfactory psychometric properties, making it suitable for use for the Greek population.

## 1. Introduction

Disgust is a basic emotion [1] that can be identified across cultures [2,3]. At first, disgust was associated with the repulsive feeling caused by the prospect of oral incorporation of an offensive object, such as rotten food or body waste products [3]. However, later studies indicated that disgust is associated not only with food rejection but also with a wide range of elicitors, such as body products, death, body envelope violations, hygiene, sex, and socio-moral violations, etc. [4]. More recently, it has been suggested that the emotion of disgust acts as a psychological mechanism that protects humans and other animals, by motivating behavioral avoidance of possible contaminants [5,6].

Disgust, as a basic emotion, has a distinct facial expression, which consists of three main components, the gape, the retraction of the upper lip, and the nose wrinkle [7]. It is also accompanied with specific physiological reaction (nausea), subjective feeling state (revulsion), and a behavioral response (keeping distance from the object of disgust) [3]. Recent neuroimaging studies suggest that the anterior insula is associated with the emotion of disgust as it was activated during the exposure of healthy participants to disgust relevant stimuli [8,9,10].

The individual ease or tendency to experience disgust more quickly and intensively is termed as disgust propensity [11]. This personality trait has been shown to predict lower willingness to approach disgust elicitors [12] and potentiate classical conditioning of disgust [13]. Moreover, disgust propensity has been shown to be positively correlated with activation of the anterior insula during exposure to disgusting stimuli [14]. Heightened levels of disgust propensity have been linked with various psychiatric disorders [15,16]. For example, it has been shown that disgust propensity is associated with contamination-based obsessive compulsive disorder [17,18] and contamination concerns [19], spider phobia [20], blood-injection-injury phobia [21], and eating disorders [22], as measured by self-report methods. Elevated disgust propensity levels have also been observed in patients with schizophrenia and depression, relative to controls [23,24]. A recent study showed that disgust propensity is associated with a negative body image and this relationship was mediated by self-disgust [25]. A widely used psychometric tool for measuring individual differences in disgust propensity is the Disgust Scale-Revised (DS-R) [26]. The DS-R is derived from the Disgust Scale (DS) and it is an improved and more reliable version of the tool [27,28]. The DS was developed by Haidt et al. [4], in order to study the emotional state of disgust, and focused on the stimuli that elicited disgust. It consists of 32 items and 8 domains of disgust elicitors, more specifically, animals, food, hygiene, magical thinking, sex, body products, death, and envelope violations. However, despite the fact that the internal consistency of the total scale was good (*α* = 0.84) [4], the eight subscales demonstrated a compromised reliability that could be attributed to the small number of items in each domain, *α*’s = 0.34–0.64 [4] and *α*’s = 0.40–0.68 [26].

Consequently, Olatunji et al. [26], after a series of analyses, suggested the reduction of the eight subscales to three (Core, Animal Reminder, and Contamination Disgust), which led to the development of DS-R. Core disgust (CO) concerns the prospective of oral incorporation of offensive items, including rotten food, small animals, and body waste products. The animal reminder disgust (AR) is related to stimuli that remind us our animal nature and mortality, such as body envelope violations and dead bodies. Lastly, contamination disgust (CD) describes the perceived threat of contagion via body byproducts and microbes [26]. The newly revised DS-R has 27 items measured on a 5-point Likert Scale (ranging from 0 to 4), 25 of them concern disgust and 2 of them are “catch” questions for identifying poor responders.

Studies that were conducted later gave additional evidence to the three factor structure [19,27,28], which was shown to have a better fit than the one or two factor (animal reminder and core-contamination) models [12,26]. Further support for the three factor model was provided by Olatunji et al. [1,19] who found evidence for a bi-factor model of disgust, which suggested that although there is a general disgust propensity factor that underlay all the items, the three DS-R factors still captures unique variance.

The DS-R scale has been validated and its three factors confirmed internationally, including a Korean sample [29], students in the Netherlands [28], samples from the United States [19,26], an Israeli sample [30], and samples from Brazil, Australia, Germany, Italy, Japan, and the Netherlands [27]. The factor structure of the DS-R has also been examined in a sample of adolescent students from the United States [31].

Previous studies have shown evidence of the influence of several demographics characteristics on DS-R scores. More specifically, disgust propensity has been reported to be higher in women than in men [4,19,27,30,31], and this finding was recently supported by an extensive meta-analysis of 90 studies from different cultures [32]. Disgust propensity has also been associated with religiosity [4,30]. A weak negative association with age has also been reported [30,33]. However, due to the restricted number of evidence, there is still need of further investigation on the influence of demographic characteristics on disgust propensity.

Given the association of disgust propensity with the etiology of some psychopathological conditions, a psychometrically sound tool measuring this construct, is of high clinical importance. In clinical practice, the tool can be used to get more insight on the symptoms, in several psychopathological conditions that have been shown to be associated with disgust (e.g., assessing whether symptoms of anxiety- and OCD-related disorders are mostly motivated by fear, distress, or disgust). As disgust has been shown to be more resistant in extinction and exposure therapy, than in fear [34], and the reduction of disgust propensity has been associated with improvement in obsessive-compulsive symptoms, after therapy [35,36], a measure of disgust propensity could also be valuable for designing (e.g., interventions that might be effective in reducing disgust [34]) and assessing treatments for anxiety and Obsessive Compulsive and related disorders. Consequently, the DS-R could be very useful in Greek clinical and research settings. However, there is no Greek version of the DS-R available yet. Therefore, the adaptation of the DS-R into Greek and the psychometric assessment of this version is very important.

The present study had four main purposes. The first aim was to assess the psychometric properties and to identify the factor structure of the translated version of the DS-R, on a sample of Greek participants. The second aim was to assess the goodness of fit of various factor structures, previously reported in the literature, including the one, two, and three factor models [26,28], and their corresponding bi-factor analogs [19]. The third aim was to evaluate the measurement invariance of the scale, with respect to age and gender. The fourth aim was to examine the influence of the demographic characteristics of our Greek sample on the DS-R scores.

## 2. Materials and Methods

### 2.1. Participants

The complete data consisted of the responses of 754 individuals originating from all 9 administrative divisions of Greece. The demographic indices of the participants are presented in Table 1. The DS-R was also re-administered in a random subset of 50 individuals in order to assess the test-retest reliability of the scale. The between-assessments interval was one month.

### 2.2. Instruments

#### 2.2.1. Demographic Characteristics

A part of the questionnaire included questions on demographics, including age, gender, occupation, education, occupation, monthly income, birthplace, religion, and religiousness. For the income, exhaustive categories were used to have a clear picture of the monthly income of our sample. Answers were categorized post-hoc into four groups of similar sample sizes (0–800€, 801–1220€, 1200–2000€, 2001+€) to enable analysis. Religiousness was assessed via a 10-point scale, in which 0 corresponded to “not at all” and 10 corresponded to “very much”.

#### 2.2.2. The Greek Version of the DS-R Scale

The DS-R scale was translated from English to Greek by a team of experienced psychologists. Then, it was translated back to English by a bilingual who is native speaker of both English and Greek, and the back translation was compared to the original. A second back translation from English to Greek was held by the team mentioned above, and was compared with the first translation. The final satisfactory version of the scale was used in the present study, according to the recommendations of Van de Vijver and Hampleton [37].

Along with the DS-R, two measurement scales were also administered to a subset of 363 individuals, in order to obtain evidence towards the concurrent validity, described below.

#### 2.2.3. The Revised Symptom Checked List (SCL-90-R)

The SCL-90-R is a self-reporting scale with 90 items which measures nine symptoms of psychopathology and psychological distress [38]. It consists of nine subscales measuring somatization, obsessive-compulsive traits, interpersonal sensitivity, depression, anxiety, hostility, phobic anxiety, paranoid ideation, and psychoticism. The Greek version of the SCL-90-R has been standardized and validated in the Greek population [39]. In the present study, its subscales had internal consistency indices between 0.78–0.88.

#### 2.2.4. The Eysenck Personality Questionnaire (EPQ)

The EPQ [40] is an 84 item scale which aims to assess the three dimensions of personality. It consists of four subscales, namely, the psychoticism (PS), the extraversion (EXT), the neuroticism (NEU), and the lie scale (LS). The EPQ has been translated in Greek and validated in the Greek population by Demetriou [41]. In the present study, the internal consistency indices of its subscales ranged between 0.60–0.84.

### 2.3. Statistical Methods

#### 2.3.1. Hypothesis Testing

For the evaluation of the strength of associations we used the Pearson’s correlation coefficient as the DS-R scores were symmetrically distributed. Differences in the means, across groups, were tested using the independent *t*-test and one-way ANOVA (more than two groups). The significance level was set to *p* < 0.05.

#### 2.3.2. Factor Structure

The sample was randomly divided into two samples, in order to apply both Exploratory Factor Analysis and Confirmatory Factor Analysis (EFA and CFA, respectively). The data were not symmetrically distributed (floor and ceiling effects occasionally occurred) and, therefore, factor analysis techniques for interval data could lead to biased estimates. Instead, factor analysis for categorical data (item factor analysis, in particular) was used in MPLUS [42], via the weighted least squares estimator (WLSMV) [43]. The dimensionality of the scale was also evaluated using the bi-factor model, according to Reise et al. [44] recommendations. The measurement invariance of the DS-R items, in relation to gender and age, was studied using the multiple indicators–multiple causes model (MIMIC) [45].

To evaluate the overall model fit in all cases, both measures of absolute and relative fit were assessed, namely, the relative chi-square (relχ^2^—values close to 2 indicate a close fit) [46], the Root Mean Square Error of Approximation ((RMSEA), values less than 0.08 are required for an adequate fit) [47], the Tucker–Lewis Index ((TLI), values higher than 0.9 are required for a close fit) [48], and the Comparative Fit Index ((CFI), values higher than 0.9 are required for a close fit) [49].

#### 2.3.3. Reliability Assessment

For the evaluation of the internal consistency of the scale and its subscales, we used the Cronbach’s alpha coefficient [50]. In order to evaluate the stability in time (test-retest reliability) we used the intra-class correlation coefficient (ICC) [51], for the agreement in the scores of the total scale and its subscales between the two time-points.

## 3. Results

### 3.1. Demographic Characteristics

Less than 1% missing data were present in our sample, confirming the good responsiveness of the scale. There were no statistical differences between genders, with respect to age (Mann–Whitney test: Z = −1.0813, *p* = 0.070, *r* = −0.067), income (Mann–Whitney test: Z = −0.487, *p* = 0.626, *r* = −0.018), occupation (χ^2^ = 4.589, df = 5, *p* = 0.468, Cramer’s *V* = 0.080), educational level (χ^2^ = 4.236, df = 5, *p* = 0.512, Cramer’s *V* = 0.076), or religiousness (Mann–Whitney test: Z = −1.310, *p* = 0.190, *r* = −0.049). However, females stated that they were Greek orthodox, more often, compared to men (92% vs. 83%, χ^2^ = 1.7, df = 1, *p* = 0.001, Cramer’s *V* = 0.123).

### 3.2. Factor Structure

#### 3.2.1. EFA Models

Six eigenvalues of the sample (polychoric) correlation matrix were larger than one (5.9, 1.8, 1.6, 1.4, 1.3, and 1.2), suggesting (according to Kaiser’s criterion) that up to six factors could be extracted. In order to investigate which model was best, we fitted all models up to six factors and the goodness of fit indices are presented in Table 2 (Crawford-Ferguson, CF-EQUAMAX oblique rotation). Even the unidimensional model had an adequate fit to our data. That is, all models in Table 2 had a good fit to our data. As the number of factors extracted increased, the fit slightly improved by default. Therefore, we could base our model choice in parsimony and face validity. The three and four factor models offered meaningful content and had a close fit to the data. Note, that the data driven EFA method results in our data were in line with the previously published ones in the literature.

The estimated factor loadings of the 2-factor and 3-factor models are presented in Table 3. According to the loadings, the items initially assigned (that is, according to Olatunji et al. [26]) to the AR load on the second factor had some cross-loadings on the third factor. The CO dimension was replicated in our data (factor 1), partially, whilst some CD items also loaded on factor 1. By restricting the factors to two, the number of cross loadings reduced. The Olatunji et al. [26] 3-factor model was replicated in our data to a satisfactory extent, but we further investigated the dimensionality of the DS-R, using CFA, in the second sample.

#### 3.2.2. CFA Models

The next step was to confirm that the previously published models fit well with our data, as well. The fit of three models was evaluated using CFA, namely:

- The unidimensional model (M_1_), where all items load on a general factor.
- The 2-factor model (M_2_), proposed by Rozin, Haidt, and McCauley [26].
- The 3-factor model (M_3_), evaluated by Olatunji et al. [26].

The models M_2_ and M_3_ were also augmented to include a general factor, in order to fit the corresponding bi-factor models (M_4_ and M_5_, respectively). In the bi-factor models the items were set to load on their designated factors and also to cross-load onto the general factor, according to the recommendations of Reise et al. [44]. Table 4 presents the goodness of fit of all models and it becomes apparent that all models show a close fit for our data.

In the remaining, we follow step-by-step, the procedure described in Reise et al. [1,19] and we compare the unidimensional model (M_1_), the 3-factor model (M_3_), and the corresponding bi-factor model (M_5_). The loadings on the factors are presented in Table 5, according to which:

(a) The item loadings on the single factor of M_1_ were similar to the loadings of the items on the general factor of the bi-factor model M_5_. According to Reise et al. [44], this indicates that there is no loss of information if the total score of DS is used.
(b) The loadings of the items on the general factor of model of the bi-factor model M_5_, were substantially larger than their loadings on the specific factors they were assigned to. According to Reise et al. [44], this indicates that the specific factors mostly reflected general disgust rather than the three factors. In fact, in some cases (items 1, 10, 15, 20, 22, 25, and 27) the loadings to the specific factors became non-significant, indicating that in the presence of general disgust, these items no longer measured specific subtraits. On the contrary, there were cases which retained the magnitude of their loadings (items 3 and 4, or even increased it (items 6 and 7). These items were specific for the corresponding factors.
(c) The loadings of the items on their designated factors in model M_3_ (where there was no general factor) was larger than their loadings to their designated factors in model M_5_ (where there was a general factor).

According to Reise et al. [44], (a), (b), and (c) suggest that both the one factor model and the three factor model could be used without loss of information. While the general factor appeared to be adequate, there was scope for presenting the three factor model, as well, according to our results.

#### 3.2.3. Measurement Invariance—MIMIC Models

The measurement invariance of the DS-R is evaluated in this section with respect to age and gender, using the MIMIC model. We estimated the direct effects of each covariate on the items. A significant direct effect from the exogenous variable (here, age and gender) to a particular item, demonstrates a lack of measurement invariance, thus, raising concerns of the measurement bias. The interpretation of the significant effects is like all models from the family of the generalized linear models, that is, the effect of each covariate is controlled for all other variables in the model. For these analyses, the complete sample and the three-factor model was used.

As age increased, it was more likely to receive higher responses in item 9, for the same expected values of underlying disgust. That is, controlled for disgust levels, the probability of agreeing with the statement “I probably would not go to my favorite restaurant if I found out that the cook had a cold”, increased with age. Significant effects were also present in the cases of items 15 (CO—maggots on a piece of meat) and 4 (CD—toilet seat), but were of negligible magnitudes (Table 6). Five items of CO and two of CD were not invariant with respect to gender. For men and women showing the same levels of disgust, men were more likely to respond positively (agreed) on items 20 (CO—ketchup on vanilla ice cream) and 25 (CO—glass of spoiled milk). Women were more likely to have increased levels of agreement with the statements on items 4 (CD—toilet seat), 9 (CD—cook had a cold), 22 (CO—change of underwear only once a week), and 27 (CO—step on an earthworm).

### 3.3. Reliability, Validity, and DS-R Scores

Cronbach’s alpha was 0.84 for the total scale reflecting satisfactory internal consistency. Table 7 presents the alpha coefficients within each of the three factors. Among the three factors, the lowest coefficient occurred for CD. However, this factor consists of only five items and the coefficient was deflated in small scales.

With respect to the test-retest reliability, the ICC ranged between 0.85 and 0.92 (*p* < 0.001 in all cases), confirming the stability of the scale at each of the three factors (Table 7). The 50 individuals, for whom the test-retest data were available, did not differ from the complete sample, with respect to their demographic and clinical characteristics (*p* > 0.05 in all comparisons).

The descriptive characteristics of the DS-R scores are presented in Table 7. The females had significantly higher scores than the males in all factors, and in the total score. The inter-correlations between the DS-R scores, as well as their associations with the demographical characteristics are presented in Table 8. Age was weakly correlated with CO. The level of education had weak negative correlations with all factors and with the total score. Positive correlations were present between the DS-R scores and the level of religiousness. One-way ANOVA was employed to test mean differences of the DS-R scores, with respect to the occupation. No statistically significant results emerged in the cases of CO (F(3.722) = 1.903, *p* = 0.128, *η*^2^*_p_* = 00.008), AR (F(3.722) = 1.739, *p* = 0.158, *η*^2^*_p_* = 0.007), and for the total DS-R scores (F(3,722) = 2.463, *p* = 0.061, *η*^2^*_p_* = 0.010). However, differences occurred in the case of CD (F(3.722) = 4.677, *p* =0.003, *η*^2^*_p_* = 0.019). According to the Bonferroni post-hoc test (multiple comparisons adjustment), differences were observed between the individuals working in the public sector (mean = 9.7) and the ones working as freelancers (mean = 8.2) (mean difference: −1.5, SE = 0.43. *p* = 0.003).

The correlations of the DS-R factors and the total score with the corresponding ones of the EPQ and the SCL-90-R scales are presented in Table 9. The total DS-R was positively correlated with the EPQ neuroticism scale, but negatively correlated with the EPQ psychoticism scale, whilst no significant correlation was found with the extraversion scale. This pattern was repeated within the DS-R factors. Only exception, the AR factor was negatively related to extraversion.

The three factor and the total score of the DS-R were significantly, positively, correlated with almost all subscales of the SCL-90-R, apart from the relationship between the AR factor and the psychoticism scale of the SCL-90-R (Table 9).

## 4. Discussion

In the present study, we evaluated the psychometric properties, the factor structure, and the measurement invariance (with respect to age and gender) of the Greek-version of the DS-R. Our results support the Olatunji et al. [26] 3-factor solution.

The EFA method concluded to a 3-factor solution with similar structure as that of the original 3-factor model, presented by Olatunji et al. [26]. Our F2 factor was almost identical to the AR factor of the 3-factor model, as all AR items loaded to this factor. However, two items cross-loaded to the other two factors. In particular, items 10 (“watch a person with a glass eye take the eye out of the socket”) and item 19 (“pick up dead cat with bare hands”) also loaded to the F3 factor (similar to the Contamination disgust factor), which might reflect the Greek sample’s association of a mutilated/deformed body with illness and a source of contamination. Moreover, there was a cross loading of item 8 (“see someone vomit”, originally in CD) with F1 (similar to CD) and F2. This finding might be attributed to the fact that in the Greek culture seeing someone vomit, is not only characterized by its offensive nature and inappropriateness for oral incorporation, but is also a reminder of the vulnerability and animal origin of humans. However, these speculations regarding Greek culture should be investigated further in future studies. Regarding the other two factors, items from both CO and CD loaded on the F1 and F3 factors, meaning that the two original factors were merged in our data. Another fact to consider is that, in this model, 6 items cross-loaded (similar or identical load) onto two factors, which might have affected the appropriateness of the model.

The 2-factor EFA solution also had an adequate fit to our data, and had fewer cross loadings than the previous solution. The present model seems to be almost identical with the two-factor model proposed by Rozin et al. in 2000 (cited in Olatunji et al. [26]), in which CO and CD formed one factor. The only difference with the proposed model was that, item 19 (“pick up dead cat with bare hands”) loaded onto the CO and CD factors, instead of the AR factor, and item 8 (“see someone vomit”) loaded onto the AR factor, instead of the other factor.

CFA was used to evaluate and compare the unidimensional model, the 2-factor and the 3-factor models of disgust, as well as their corresponding bi-factor models. Contrary to previous studies [26,28] in which the three-factor model had a superior fit to the one and two-factor models, all models were shown to have a close fit to the data, with no substantial differences between them. The bi-factor models had a slightly better fit to the data, in accord with Olatunji et al. [19].

In the bi-factor analyses, all loadings of the items on their specific factors were reduced, nine of them became non-significant after the addition of the general factor of disgust, and most of them were smaller than the loadings on the general factor. This finding might be explained by the general and broad nature of core disgust [3,7]. Nevertheless, four items either retained the magnitude of their significant loadings (item 3, CO, item 4, CD) or increased it (item 6, CD, item 7, AR), after the introduction of the general factor to the model, indicating that these items were specific to the corresponding domains of disgust. In conclusion, according to the recommendations of Reise et al. [44], the bi-factor analyses suggests that there is no loss of information if the DS-R is used as a unidimensional measurement scale via its total score, but at the same time the three-factor solution has a good fit to the data and can also be applied. Our results are partially in line with the ones reported by Olatunji et al. [19], but the importance of the general disgust factor might be more pronounced in the Greek population and this could be explained by its culture and the everyday practices for avoiding disgusting objects. As suggested by one of the reviewers, the scale would potentially be benefited by appropriately revising the set of items, to empower the multidimensionality aspect.

Our finding regarding the strength of the general factor of disgust underlines its importance in disgust propensity. A common characteristic of most disgust elicitors is their animal nature. They are of animal origin as they might be animals (small animals that elicit disgust), animal byproducts, or reminders of our animal nature and vulnerability [3]. However, to the best of our knowledge, this is the first study showing the superiority of the general factor, over the three factor model. This finding was less pronounced in the first study of Olatunji et al. [19], who also applied a bi-factor model to their data. As a result, no safe assumptions can be made regarding the unidimensionality of disgust, yet, as this finding could be unique in the Greek population. Future research could evaluate the bi-factor model of disgust in other countries, to examine how the importance of the general disgust factor to the total variance, differs across cultures

The measurement invariance testing, with respect to gender and age revealed that there are considerations of measurement bias. Specifically, the present study suggests gender non-invariance in 6 items. Items 4, 9, 22, 27 (2 CD, 2 CO) were more likely to be endorsed by women. Whereas, items 20 and 25 (CO) were stronger indicators for men. The reason for this was still unclear. However, all four items that women agreed on more, described possible contaminants, and both items that men agreed on more, described possible oral incorporation of inappropriate foods. Kim et al. [31] also found gender non-invariance, though in different items. However, the items that adolescent boys scored higher on, described food products—a finding that is in partial agreement with the present study. Finally, the present study also revealed that item 9 (CD) and less profoundly items 15 and 4, were more likely to have larger scores, as age increased. To our knowledge, this was the first study to examine measurement invariance in the DS-R item, with respect to age. More research is needed to examine measurement invariance of the DS-R, regarding the different demographic factors.

All reliability indices of the scale were satisfactory. The test-retest reliability indices confirmed the stability of the scale. The Cronbach’s alpha of the total scale and the CO and AR subscales were high, showing a satisfactory internal consistency. However, the internal consistency of the CD subscale was relatively low, being in line with previous findings [26,28,29], and this could be attributed to some extent to the fact that, there was a small number of items in this factor. This could be addressed by increasing the number of items in the contamination subscale or adopting fewer factor solutions. All inter-correlations between the three factors of the DS-R were strong and statistically significant.

The present study also assessed the influence of demographic characteristics (religiousness, education, employment, income, gender, and age) on DS-R scores. First, religiousness was associated with the mean scores of all disgust factors, indicating that more religious participants tended to report higher levels of disgust. To our knowledge, this was the first study in which disgust propensity was assessed in an eastern orthodox sample. This finding was in accord with previous studies in which religiousness was associated with heightened levels of disgust [4,52]. Moreover, there was a weak negative correlation between levels of disgust and years of education, which contradicted the finding of Berger et al. [30], in which disgust level was independent of education. Income was also independent of disgust, showing that it was unaffected by the financial and social status.

The present study also showed that women scored significantly higher than men in all DS-R factors and on the total scale. More pronounced disgust propensity in females than in males was also found in previous studies [27,29,53], while a recent one revealed a modest female superiority, over males, in recognizing facial disgust cues [54]. Heightened levels of disgust in women might be explained by the compensatory behavioral prophylaxis hypothesis [55]. This hypothesis states that heightened disgust was employed in response to the compromised immune system, during the luteal phase of the menstrual cycle (when the body was prepared for possible pregnancy) and the early stages of pregnancy [55,56]. A recent series of two studies had also suggested that this difference in disgust between the two genders might be explained by risk-taking differences, where males were more willing to take risks than women, to attain some goals, and this might include getting exposed to pathogens [32]. These remain to be further investigated by future studies.

Finally, the present study revealed a weak but significant correlation between age and Contamination Disgust, but not with the other subscales or the total score. This finding contradicts previous research in which it was shown that disgust propensity was less pronounced in the elderly [30], as emotion control seemed to be improved with age [57]. More research is needed to evaluate the development of disgust propensity and reactivity, across the life span.

Consistent with the findings of Haidt et al. [4], examining the original DS, the total DS-R score was positively correlated with the EPQ neuroticism subscale, and negatively correlated with the EPQ psychoticism subscale. The correlation coefficients, though weak, were of similar magnitude with those observed by Haidt et al. [4] and Kang et al. [29], providing evidence of concurrent validity of the Greek version, related to personality correlates. Τhe total score of the DS-R was also positively correlated with all subscales of SCL-90, a finding that is in line with previous research in which the DS [4] was used [58]. However, these correlations were all weak (varied between 0.2 and 0.3) but of similar magnitude to those reported previously [58].

Regarding the discriminant validity, there was no correlation between the extraversion subscale and the total DS-R score, but there was a weak significant negative correlation between this subscale and the Animal Reminder mean score. However, there was an unpredicted but weak correlation between the Lie subscale and the total DS-R score, which was inconsistent with previous research [4,29]. This might suggest a possibly compromised discriminant validity of the Greek DS-R, as self-presentation concerns might have affected the responses. Nevertheless, given that the correlation was weak, this limitation was restricted. Future research could use additional measures, such as the Self-Monitoring Scale by Snyder (1997), as cited in [4], in the Greek DS-R, to further assess discriminant validity.

The present study had several limitations. Due to the lack of appropriate standardized psychometric tools relevant to the construct of disgust and OCD symptomatology in the Greek population, the assessment of the convergent validity of the Greek version of the DS-R was not feasible at this time point. The DS-R was the first measurement of disgust that has been validated in the Greek population, no other psychometrically assessed measurement exists. Moreover, the association between the DS-R and the OCD symptomatology, which could be examined as an indication of convergent validity, as in previous studies, [26], was assessed only by the OCD subscale of the SCL-90, which did not allow the discrimination of OCD symptoms types (e.g., Contamination Concerns versus Checking). However, there are no published validation of an OCD scale in the Greek population available for use, yet, so such a measurement could not be used in the present study.

The present results lay the foundation for further research. For example, future study could evaluate the predictive validity of the Greek DS-R and its domains, by using behavioral avoidance and physiological tasks, e.g., [53]. Τhe DS-R could also be administered in Greek participants that have been diagnosed with disorders associated with an elevated disgust propensity, such as obsessive-compulsive disorder, blood-injection injury, and small animal special phobias [15]. The DS-R scale, alongside other measures of disgust, could also be used to assess disgust propensity in patients with an acute onset of Obsessive-Compulsive symptoms, which could be explained by inflammatory, infective, and immunological conditions, such as ‘Pediatric Autoimmune Neuropsychiatric Disorders Associated with Streptococcal Infections’ (PANDAS) [59]. The Greek version of the DS-R could also be used to adapt and validate other scales of disgust in the Greek population, which could be useful in clinical settings. An example of these scales is the Propensity and Sensitivity Scale (DPSS) [60], which assesses—in addition to the disgust propensity—disgust sensitivity (negative appreciation of experiencing disgust), which have also been associated with psychopathology [61,62]. Finally, given that our results outlined the strength of the general factor of digust, future research could assess the dimensionality of the scale, by using the bi-factor model, in participants from various cultures, and further revising the scale to improve its structure.

## 5. Conclusions

The present study showed satisfactory psychometric properties for the Greek version of the DS-R, making it suitable for use in the Greek population, for both clinical and research work. Given the relatively weak, but significant associations between the DS-R and all subscales of the SCL-90, and our previous findings, the respective scale might be useful while assessing symptoms of various psychopathological conditions, including OCD with contamination concerns, anxiety disorders, affective disorders, and schizophrenia in Greek clinical settings. However, more research with clinical samples is needed. Moreover, the present study showed some evidence of the three-factor solution of the Greek version of the scale. However, it was revealed that the general factor of disgust was stronger and future research should address this issue of dimensionality.

## Figures and Tables

**Table 1:** Demographic characteristics of the sample.

**Gender**	Males: 254 (34%)		Females: 492 (65%)					Missing: 8 (1%)
**Age**	Mean: 37		SD: 12	Range: 18–78				Missing: 23
**Occupation**	Public sector: 226 (30%)		Private sector: 196 (26%)	Freelancer: 121 (16%)		Non-employed (pensioners, students, unemployed): 184 (24%)	Pensioners: 32 (4%)	Missing: 28 (4%)
							Students: 104 (14%)	
							Unemployed: 47 (6%)	
**Education**	1st level: 9 (1%)		2nd level: 165 (22%)	3rd level: 574 (76%)	Bachelor: 397 (53%)			
					M.Sc.: 145 (19%)			
					PhD: 32 (4%)			
**Income**	0–800€: 138 (18%)	0–400€: 53 (7%)	801–1200€: 193(26%)	1200–2000€: 185 (25%)		2001€+: 212 (28%)	2001–2400€: 69 (9%)	Missing: 26 (3%)
		401–800€: 85 (11%)					2401–2800€: 40 (53%)	
							2801€+: 103 (14%)	
**Religion**	Greek orthodox: 633 (84%)		No religion: 74 (10%)	Other: 6 (1%)				Missing: 41 (5%)
**Religiousness**	Mean: 4.5		SD: 2.9	range: 0–10				Missing: 28
**Birthplace**	Region		Ν	%				
	Epirus		31	4.1				
	Thessaly		26	3.4				
	Thrace		8	1.1				
	Crete		27	3.6				
	Macedonia		42	5.6				
	Aegean islands		46	6.1				
	Ionian Island		17	2.3				
	Peloponnese		78	10.3				
	Central Greece		438	58.1				
	Missing		41	5.4				

**Table 2:** Goodness of fit indices per model—Exploratory Factor Analysis (EFA) sample.

Model	Relative χ^2^	RMSEA	TLI	CFI
1-factor	2.4	0.062	0.82	0.84
2-factors	2.2	0.056	0.86	0.88
3-factors	2.0	0.050	0.88	0.91
4-factors	1.7	0.044	0.91	0.94
5-factors	1.6	0.041	0.92	0.95
6-factors	1.5	0.035	0.94	0.97

Note: RMSEA = Root Mean Square Error of Approximation; TLI = Tucker–Lewis Index; CFI = Comparative Fit Index.

**Table 3:** Rotated (oblique Crawford-Ferguson, CF-Equamax) factor matrix of the Disgust Scale-Revised (DS-R) scales—EFA Sample (*n* = 378).

DS-R Item	Original 3-Factor	2-Factor Model		3-Factor Model		
		F1	F2	F1	F2	F3
2. see a human hand preserved in a jar	AR	0.53			0.50	
5. walking through a graveyard	AR	0.59			0.54	
7. touch a dead body	AR	0.63			0.59	
10. watch a person with a glass eye take the eye out of the socket	AR	0.33			0.31	0.26
14. sleep in a hotel room where a man had died of a heart attack	AR	0.37			0.32	
19. pick up dead cat with bare hands	AR		0.31		0.25	0.31
21. see a man with his intestines exposed	AR	0.62			0.62	0.26
24. touch the ashes	AR	0.52	0.27		0.49	0.43
1. eating monkey meat	CO		0.28			0.30
3. clear a throat full of mucous	CO		0.33	0.55		
6. cockroach in someone else’s house	CO	−0.24	0.40			0.41
8. see someone vomit	CO	0.31		0.35	0.27	
11. see a rat in a park	CO		0.30			0.23
13. soup stirred by a used but thoroughly washed flyswatter	CO		0.36	0.52		
15. see maggots on a piece of meat	CO		0.42			0.35
17. smell urine in a tunnel	CO		0.60	0.37		0.35
20. put ketchup on vanilla ice cream	CO		0.33			0.42
22. friend changes underwear only once a week	CO		0.57	0.31		0.41
25. drink a glass of milk when you smell that it is spoiled	CO		0.42			0.43
27. step on an earthworm	CO		0.47			0.42
4. my body touch the toilet seat	CD		0.40	0.53		
9. the cook had a cold	CD		0.21	0.32		
18. drank from the glass that an acquaintance had been drinking from	CD		0.56	0.36		0.36
23. chocolate shaped like dog-doo	CD		0.50			0.41
26. inflate a new unlubricated condom, using your mouth	CD		0.54			0.51

Rotation: Oblique CF Equamax. Note: DS-R = Disgust Scale- Revised, AR = Animal Reminder, CO = Core Disgust, CD = Contamination Disgust.

**Table 4:** Goodness of fit indices—Confirmatory Factor Analysis (CFA) sample.

	Relative χ^2^	RMSEA	TLI	CFI
Unidimensional model	1.9	0.049	0.93	0.93
2-factors	1.8	0.047	0.93	0.94
2-factors-bi-factor	1.8	0.045	0.94	0.95
3-factors	1.8	0.047	0.93	0.94
3-factors-bi-factor	1.8	0.045	0.94	0.95

Note: RMSEA = Root Mean Square Error of Approximation; TLI = Tucker-Lewis Index; CFI = Comparative Fit Index.

**Table 5:** Confirmatory Factor Analysis loadings for the unidimensional, the 3-factor, and the bi-factor 3-factor model.

DS-R Item	1-Factor Model (Μ_1_)	3-Factor Model (Μ_3_)			Bi-Factor 3-Factor Model (Μ_5_)			
	General Factor	CO	AR	CD	General Factor	CO	AR	CD
1	0.37 **	0.37 **			0.38 **	−0.07 ^#^		
2	0.40 **		0.42 **		0.36 **		0.23 **	
3	0.39 **	0.40 **			0.38 **	0.37 **		
4	0.26 **			0.27 **	0.29 **			0.35 *
5	0.57 **		0.57 **		0.49 **		0.31 **	
6	0.19 **	0.20 **			0.18 ***	0.28 ***		
7	0.57 **		0.60 **		0.49 **		0.57 **	
8	0.54 **	0.56 **			0.54 **	0.40 **		
9	0.43 **			0.44 **	0.43 **			0.09 ^#^
10	0.23 **		0.24 **		0.22 **		0.07 ^#^	
11	0.43 **	0.44 **			0.43 **	0.23 **		
13	0.51 **	0.52 **			0.53 **	−0.26 **		
14	0.63 **		0.66 **		0.57 **		0.36 **	
15	0.56 **	0.58 **			0.57 **	0.04 ^#^		
17	0.62 **	0.63 **			0.64 **	−0.14 *		
18	0.51 **			0.53 **	0.51 **			0.20 *
19	0.58 **		0.61 **		0.56 **		0.17 *	
20	0.48 **	0.49 **			0.49 **	−0.09 ^#^		
21	0.58 **		0.61 **		0.56 **		0.20 *	
22	0.52 **	0.53 **			0.53 **	0.05 ^#^		
23	0.68 **			0.70 **	0.68 **			0.14 ^#^
24	0.74 **		0.78 **		0.71 **		0.24 **	
25	0.42 **	0.43 **			0.42 **	0.13 ^#^		
26	0.66 **			0.68 **	0.66 **			0.35 **
27	0.52 **	0.53 **			0.53 **	0.01 ^#^		

Note: DS-R = Disgust Scale-Revised, AR = Animal Reminder, CO = Core Disgust, CD = Contamination Disgust, * *p* < 0.05, ** *p* < 0.01, *** *p* < 0.001, # *p* > 0.1.

**Table 6:** Multiple indicators–multiple causes (MIMIC)—indirect effects for age and gender.

	Item	Direct Effect	*p*-Value
age	(CO) 15	0.011	<0.001
	(CD) 04	−0.009	0.026
	(CD) 09	0.288	0.001
gender	(CO) 20	0.433	<0.001
	(CO) 22	−0.334	<0.001
	(CO) 25	0.427	<0.001
	(CO) 27	−0.248	0.003
	(CD) 04	−0.272	0.006
	(CD) 09	−0.011	0.002

*Note:* AR = Animal Reminder, CO = Core Disgust, CD = Contamination Disgust.

**Table 7:** Reliability and descriptive indices of Disgust Scale (DS).

	Reliability		DS Scores							
	*α*	ICC (95% CI)	Males (Ν = 254)		Females (Ν = 492)		Independent samples *t*-test	Total (Ν = 754)		Effect size
			*M*	*SD*	*M*	*SD*	t (df)	*M*	*SD*	*d*
CO	0.70	0.9 (0.82, 0.94)	29.1	7.4	24.8	7.5	−7.42 (744) ***	27.6	7.7	0.6
AR	0.72	0.92 (0.86, 0.95)	18.0	6.5	15.1	6.1	−5.75 (744) ***	17.0	6.5	0.5
CD	0.54	0.85 (0.75, 0.92)	9.4	3.9	8.1	3.5	−4.53 (562.6) ***	8.9	3.8	0.4
Total DS-R	0.84	0.92 (0.93, 0.98)	6.3	15.3	51.9	14.2	−7.23 (744) ***	57.4	15.5	0.6

Note: DS-R = Disgust Scale-Revised, AR = Animal Reminder, CO = Core Disgust, CD = Contamination Disgust, *α* = Cronbach’s alpha coefficient, ICC = intra-class correlation coefficient, CI = Confidence Interval *** *p* < 0.001.

**Table 8:** Pearson Correlation Coefficients between Demographic Characteristics and DS-R Domains.

	CO	AR	CD	Total DS
AR	0.6 ***	-	-	-
CD	0.6 ***	0.5 ***	-	-
Total DS	0.9 ***	0.8 ***	0.8 ***	-
Age	0.0 ^#^	0.0 ^#^	0.1 ***	0.0 ^#^
Income	0.0 ^#^	0.0 ^#^	0.0 ^#^	0.0 ^#^
Education	−0.1 **	−0.1 *	−0.1 **	−0.1 **
Religiousness	0.2 ***	0.2 ***	0.2 ***	0.2 **

Note: DS-R = Disgust Scale- Revised, AR = Animal Reminder, CO = Core Disgust, CD = Contamination Disgust. * *p* < 0.05, ** *p* < 0.01, *** *p* < 0.001, # *p* > 0.1.

**Table 9:** Pearson correlation coefficients between the factors of the DS-R, the Eysenck Personality Questionnaire (EPQ), and the Revised Symptom Checked List (SCL-90-R).

		CO	AR	CD	Total DS-R
EPQ	EXT	0.0 ^#^	−0.1 **	−0.1	−0.1
	NEU	0.2 ***	0.2 ***	0.2 ***	0.2 ***
	PS	−0.2 ***	−0.1	−0.1 *	−0.2 ***
	LS	0.1 **	0.0 ^#^	0.2 ***	0.1 **
SCL-90-R	ANX	0.2 **	0.2 **	0.2 ***	0.2 ***
	DEP	0.2 **	0.1 *	0.2 **	0.2 **
	PA	0.1 *	0.2 ***	0.2 ***	0.2 ***
	HOS	0.1 *	0.1 *	0.2 **	0.2 **
	IS	0.2 ***	0.2 ***	0.2 ***	0.3 ***
	OCD	0.2 ***	0.2 ***	0.3 ***	0.3 ***
	PI	0.2 ***	0.2 ***	0.3 ***	0.3 ***
	PSY	0.2 **	0.1	0.2 ***	0.2 **
	SOM	0.1 *	0.1 *	0.2 ***	0.2 **

Note: CO = Core Disgust, AR = Animal Reminder, CD = Contamination Disgust, EPQ = Eysenck Personality Questionnaire, EXT= Extraversion, NEU= Neuroticism, PS= Psychoticism, LS = Lie, SCL-90 –R = revised Symptom checked list, ANX = Anxiety, DEP = Depression, PA = Phobic Anxiety, HOS = Hostility, IS = Interpersonal Sensitivity, OCD = Obsessive Compulsive Disorder, PI = Paranoid Ideation, PSY = Psychoticism, SOM = Somatization. * *p* < 0.05, ** *p* < 0.01, *** *p* < 0.001, # *p* > 0.1.
